# Book Notices

**Published:** 1890-11

**Authors:** 


THE NEUROSES OF THE GENITO-URINARY SYSTEM IN THE MALE, WITH STERILITY AND IMPOTENCE. By Dr. R. Utzmann, Professor of the Genito-Uurinary Diseases, Viruna.

Translated by G. W. Allen, M. D., Boston. Pp. 160. Price $1.00 Philadelphia: F. A. Davis, publisher; 1890.

Any one taking the trouble to read this little book, will find it interesting and valuable. B.

---

HOW TO PRESERVE HEALTH.—By Louis Borkan, M. D. All rights reserved. The trade supplied by the American News Company, New York, 1890.

---

MEDICAL CONSULTATION BOOK. A Pharmaceutical and Clinical Book of Reference—containing the therapeutics of a full list of the officinal and non-officinal articles of the materia medica, with a consideration of the action of medicines; including an extensive collection of favorite prescriptions from the most reliable authorities of the medical profession; and all so classified as to be of ready access for authenticated treatment of each disease in the different stages and complications. Designed for the consultation room. By G. P. Hachenburg, M. D., Austin, Texas.

We have examined the manuscript of the above-mentioned work which Dr. Hachenburg intends publishing shortly. Its scope is most complete and comprehensive, and it cannot fail to serve a very valuable purpose as a book of general reference. The preparation of the manuscript shows a world of painstaking care and evinces deep research. and a familiarity with the subject treated that is rarely found in the general practitioner. Dr. Hachenburg is an old U. S. army surgeon. Agents wanted in Texas.

---

THE PHARMACOLOGY OF THE NEWER MATERIA MEDICA, embracing the Botany, Chemistry, Pharmacy and Therapeutics of new remedies. Being the results of the collective investigation of new remedies under the "working bulletin" system. Properly arranged, classified, indexed and placed at the disposal of the medical profession. Issued in monthly parts. Subscription price $2 in advance, single copies 25 cents each. Geo. S. Davis, Publisher, Detroit, Mich.

We have received parts I. to V. Part V. contains an exhaustive treatise on Coca Leaves, and is alone worth the price of the set. Messrs. Parke, Davis & Co., the pioneers in new remedies, have placed the profession under additional obligations by the publication of this valuable work, which is the outcome of the patient diligent and and intelligent investigation that has been going on in their laboratory for the past few years. For further information address the publisher.

---

PRACTICAL SANITARY AND ECONOMIC COOKING adapted to persons of moderate and small means, by Mrs. Mary Hinman Abel. The *Lomb Prize Essay*. Pp. 190.

Published by the American Public Health Association, 1890, Rochester, N. Y. Any work that teaches how to cook economically and well is valuable. This is a valuable work. B.

---

ESSENTIALS OF THE DISEASES OF THE EYE, NOSE AND THROAT. By Drs. Jackson and Gleason, of Philadelphia. 1890.

This is another of Saunder's question compends and contains the essentials, as far as it goes, of the subject treated. This about expresses it—*as far as it goes*. There is such a thing as boiling subject matter to death. One arrives at this conclusion from reading some of these essentials. B.

---

A TREATISE ON MATERIA MEDICA, PHARMACOLOGY AND THERAPEUTICS. By John V. Shoemaker, A. M., M. D., Professor of Materia Medica, Pharmacology and Therapeutics in the Medico-Chirurgical College of Philadelphia. In two volumns. Vol. 1 devoted to Pharmacy, General Pharmacology and Therapeutics, and Remedial Agents not properly classed with drugs. Philadelphia and London: F. A. Davis, publisher. 1889.

Part I. is devoted to Materia Medica, Pharmacy, Pharmacolo- and Therapeutics. Part II. to remedies and remedial agents in the treatment of diseases not properly classed with drugs.

The work shows that much pains has been taken in its preparation. The classification and arrangement are based upon the modern idea of Therapeutics. The discussion of subjects is clear and comprehensive. Vol. II. is anxiously looked for. B.

ESSENTIALS OF DISEASES OF THE SKIN. By Henry W. Stellwagon, M. D., Attending Physician to the Philadelphia Dispensary for Skin Diseases, etc. Philadelphia: W. B. Saunders. 1890.

This little book is No. 11 of Saunders' Question-Compend Series, and is arranged in ihe form of questions and answers. While a great deal can be put on a page in such a form, the physician is usually disappointed when he attempts to search for any particular point. B.

---

ELECTRICITY IN THE DISEASES OF WOMEN, with Special Reference to the Application of Strong Currents. By G. P. Massey, M. D. Second edition, revised and enlarged; pp. 240. Price, $1.50. Philadelphia: F. A. Davis, publisher. 1890.

There is not a great deal that is new in this edition of Dr. Massey's book, but quite enough .hat is interesting to justify its purchase and perusal. B.

